# Imaging cell spheroid clusters: An MRI protocol for non-invasive standardized characterization

**DOI:** 10.1016/j.heliyon.2025.e41803

**Published:** 2025-01-17

**Authors:** Rebecca Wißmann, Petros Martirosian, Marina Danalache, Gerd Grözinger, Fritz Schick, Stefanie Elser

**Affiliations:** a Department of Diagnostic and Interventional Radiology, University Hospital of Tübingen, D-72076, Tübingen, Germany; b Section on Experimental Radiology, Department of Diagnostic and Interventional Radiology, Tübingen University Hospital, Tübingen, Germany; c Department of Orthopaedic Surgery, University Hospital of Tübingen, D-72076, Tübingen, Germany

**Keywords:** Spheroids, Magnetic resonance imaging, Visualization, 3D culture, Tumor model, Regenerative medicine, Mesenchymal stem cells, Extracellular matrix, Microenvironment

## Abstract

Over the past decade, significant progress has been made in the utilization of three-dimensional cell cultures in the form of spheroids as a bridge between *in vitro* and *in* vivo models. This is contributed by natural cell-cell interactions that occur within spheroids, leading to the subsequent development of extracellular matrix. The assessment of cell spheroids with conventional microscopy is destructive, requiring sectioning that damages their micro-structures. To address these issues, we developed and propose a non-invasive approach using magnetic resonance imaging (MRI). Despite its limited spatial resolution, this method adeptly reveals information about the composition and vitality of stem cell and cancer spheroids and their micro-environment in a non-invasive manner. This work reports on the development of an MRI-compatible setup for culturing cell spheroids, tailored for use with standard 3 T whole-body MRI systems. Systematic studies with different cell types show the potential of the proposed approach for simultaneous actuation and visualization of cell spheroids, with potential across a broad spectrum of applications.

## Graphical abstract

**Figure:**
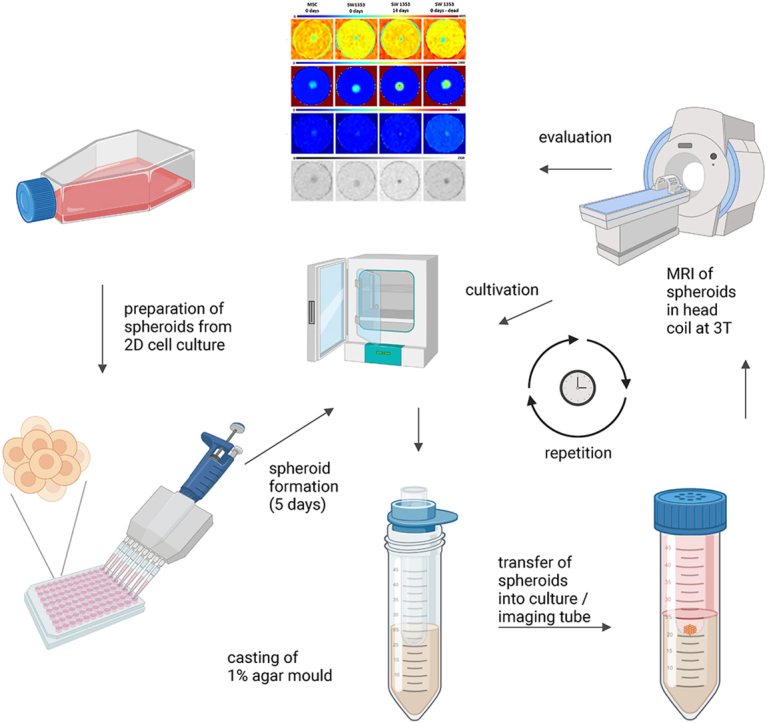

## 1 Introduction

Three-dimensional (3D) cell culture in the form of spheroids has become a prominent area of research in the field of preclinical and regenerative medicine. In comparison to conventional two-dimensional cultivation techniques, the growth of cells in a more physiologically relevant (micro-) environment has been demonstrated to exert a significant influence on cellular behavior, including proliferation, differentiation and metabolism [[1], [2], [3]]. This is associated with the presence of robust homophilic interactions of E-cadherins, which enable tight cell-cell adhesions [4]. Moreover, a dense extracellular matrix (ECM) develops over time, primarily consisting of fibrous structural proteins (such as collagen, elastin, and fibrillin), glycoproteins (e.g. fibronectin) and proteoglycans (e.g. glycosaminoglycan). These ECM components are linked to cells by means of integrins [5,6]. These intricate cell-cell adhesions and cell-to-matrix interactions culminate in the formation of gradients for nutrients, gases, growth factors, and signaling factors [[7], [8], [9], [10]]. Consequently, spheroids exhibit a distinctive three-layered structure: an outer proliferative rim with direct access to nutrients, a central zone of quiescent cells with minimal metabolic activity, and a necrotic core where nuclei disintegrate due to nutrient deprivation and the accumulation of toxic waste products [11,12].

The heterogeneous cellular composition resulting from the distribution of oxygen and nutrients and the formation of the extracellular matrix (ECM) resembles that of avascular tumors. Consequently, spheroids have become a well-established *in vitro* tumor model. In accordance with their morphological characteristics, the gene profile is analogous to that observed in solid tumors *in vivo* [[13], [14], [15]], thus rendering them a valuable instrument in the context of therapeutic screening, including drug testing, radiotherapy and immunotherapy research [[16], [17], [18], [19]]. Spheroids can therefore serve as a conduit between cell-based two-dimensional assays and animal studies [20]. The enhanced robustness of spheroids in comparison to single-cell suspensions has rendered them a favored choice in the field of regenerative medicine. In particular, stem cell spheroids have demonstrated superior viability upon application [21,22]: The use of mesenchymal stem cell (MSC) spheroids has become a standard technique for the regeneration of damaged cartilage in the context of osteoarthritis [23,24]. This approach could also be applied to other defective tissues, such as those affected by peripheral arterial disease (PAD), as demonstrated in an *ex vivo* animal model in which MSC spheroids were applied intravascularly [25].

The current mainstay of spheroid visualization is optical microscopy. The characterization of size, morphology and internal organization has been described using bright-field [26], dark-field [27], phase-contrast [28] and fluorescence microscopy [29]. For comprehensive analysis of cellular spheroid morphology at high magnification, including insights into cell-cell adhesions, scanning electron microscopy (SEM) has been demonstrated to be an invaluable tool [29,30]. Although these techniques permit comprehensive visualization of specimens, they are unable to provide a complete cross-sectional view of intact spheroids. Cross-sectional imaging typically relies on histological sectioning, which ultimately compromises the integrity of the spheroid, often resulting in deformation or fracturing [31]. Furthermore, the monitoring of cells within scaffolds can prove challenging due to the limitations of low resolution and poor focus, which are often associated with specimen thickness.

The quantitative evaluation of MRI characteristics of a sample enables an assessment of its properties to be made without causing any disruption. To date, the majority of MRI studies on spheroids have been conducted using high-field scanners (7T and 12T), with the spheroids embedded in hydrogels or labelled with contrast agents [[32], [33], [34]]. The objective of this study was to develop an appropriate cultivation tube for cell spheroids that can simultaneously function as a setup for 3T systems which are commonly used in the clinic. Those MRI systems provide high magnetic field homogeneity and sufficient spatial resolution to enable non-invasive characterization of cell aggregates.

Modern MRI techniques comprise pixel-by-pixel mapping of T1 and T2 values, apparent diffusion coefficients (ADC), and magnetization transfer ratios (MTR) to provide information about tissue composition and characteristics. The relaxation times T1 and T2 are dependent on the composition of the intra- and extracellular compartments of the specimen. This includes the presence of ions and molecules of varying size, structure, and mobility, as well as their interaction with water molecules. The latter are primarily responsible for generating the recorded MR signal [35]. Diffusion-weighted imaging (DWI) with assessment of the ADC is sensitive to obstacles to Brownian motion, such as cell membranes, and thus can be employed to detect changes in cell size, integrity, or viability [36]. MTR is correlated with the interaction of measurable mobile water and macromolecules (e.g. through the exchange of protons or the exchange between the bound water in macromolecular shells and free water) [37].

The initial phase of this project entailed the development of an MR-compatible setup for the repeated MRI study of native cell spheroids, while concurrently facilitating their appropriate cultivation without the necessity for translocation, thus avoiding any disruption to their spatial structure. Furthermore, it is essential to attain a high degree of homogeneity of the magnetic field within the sample and to guarantee the sensitive recording of the MR signal by standard receiver coils situated in close proximity to the sample. The utilization of a 3-T whole-body system permits optimal access and straightforward handling, in comparison to high-field imaging systems [38,39].

Furthermore, systematic studies were conducted with spheroids from different cell types to test the capabilities of the MR-compatible set-up and some quantitative MRI examination methods. MRI has the advantage of obviating the time-consuming preparation of histological samples. Furthermore, it may be advantageous in situations where source material is limited, as is often the case with autologous stem cell derivation, as these cells can be continuously cultured. The capacity for repeated imaging enables a targeted set of observations to be conducted at designated, pre-determined time points throughout the process of cellular development.

## 2 Materials and Methods

### 2.1 Cell culture

Human bone marrow-derived MSCs were isolated at the University Hospital Tübingen with written informed consent from the patients and approval from the local ethics committee (approval number: 401/2013BO2, date: August 27, 2013). The cells were isolated in accordance with the methodology described by Battula et al. [50]. The MSCs were cultivated in Alpha-MEM (Gibco/Thermofisher, Waltham, MA, USA) supplemented with 10 % (v/v) fetal calf serum (Gibco/Thermofisher, Waltham, MA, USA) and 1 % (v/v) penicillin-streptomycin (10.000 U/ml Gibco, Thermo Scientific, Waltham, MA, USA) at 37 °C in a humidified atmosphere containing 5 % CO_2_. For the production of 3D MSC spheroids, cells in the third to fifth passage were utilized. Once the cells reached 80 % confluence, they were detached and counted using a cell counter.

Human SW1353 chondrosarcoma cells (American Type Culture Collection (ATCC), Virginia, USA, HTB-94) were cultivated in DMEM: Ham's F12 (Gibco/Thermofisher, Waltham, MA, USA) + 10 % (v/v) FCS + 1 % (v/v) penicillin streptomycin at 37 °C under the same conditions as the MSCs. After reaching 80 % confluence, the cells were detached and counted with a cell counter.

### 2.2 Production and size distribution of 3D cell spheroids

In order to generate three-dimensional spheroids, cells were seeded into low-attachment 96-well plates (Thermo Scientific, Waltham, MA, USA) at densities of 62,500 cells per well. Following the seeding and incubation of the cells for a period of five days under standard cell culture conditions (37 °C, 5 % CO_2_, normoxia), the cells exhibited growth in a three-dimensional configuration, forming stable spheroids.

### 2.3 Preparation of cultivation tubes

A 1 % (w/v) agarose gel (Thermo Scientific, Waltham, MA, USA) was prepared with phosphate buffered saline (PBS, Gibco/Thermofisher, Waltham, MA, USA) and subjected to autoclaving at 121 °C and 1 bar. Consequently, 20 ml were transferred to a 50 ml tube (Greiner Bio-One International GmbH, Kremsmünster, Austria). A smaller, sterile tube (14 ml, Greiner Bio-One International GmbH, Kremsmünster, Austria) was employed as a mold to create a small denture (approximately 1 cm deep) on the surface for spheroid placement. For the purposes of cultivation, the lid was replaced with a ventilation lid of a 175 cm^2^ cell culture flask. Prior to their transfer into the cultivation tubes and after a five-day period of spheroid formation, the cells were subjected to a preheating process in a water bath at a temperature of 37 °C. A total of 192 spheroids were utilized for each cultivation tube. Approximately 30 ml of the respective media was added and replaced twice per week.

### 2.4 Creation of dead cells with methanol

The freshly formed spheroids were transferred to the cultivation tubes, and the existing medium was removed. Subsequently, the spheroids were washed twice with PBS. In order to maintain cellular integrity, the spheroids were covered with ice-cold methanol (at a temperature of −20 °C, Merck KGaA, Darmstadt, Germany) and incubated at −20 °C for a period of 1 h. Subsequently, the methanol was removed, and the cells were washed three times with PBS. In order to standardize the experimental setup, the cells were subsequently immersed in culture medium and maintained under typical cell culture conditions.

### 2.5 Preparation of histological sections and H&E staining

Spheroids were collected and fixed in 4 % formaldehyde (Otto Fischar GmbH & Ko KG, Saarbrücken, Germany) before being washed with PBS. Subsequently, the specimens were pre-embedded in 2 % (w/v) agar to prevent the spheroids from accumulating at the bottom of the histology cassette. Subsequently, the embedded blocks were sectioned at a thickness of 4 μm using a microtome, transferred to glass slides, and allowed to dry in air. H&E staining was performed using Mayer's hematoxylin and eosin Y solution (Diapath, Martinego, Italy).

### 2.6 Fluorescence staining and imaging

For fluorescent staining, the spheroids were collected and transferred into a 96-well plate. Dual staining of live and dead cells was conducted by incubating the spheroids with 10 μg/ml propidium iodide (Thermo Scientific, Waltham, MA, USA) and 4 μM calcein AM (Cayman Chemical Ann Arbor, MI, USA) for 20 min. Fluorescence imaging was performed using a Leica DMi8 microscope (Leica, Wetzlar, Germany).

### 2.7 MR imaging

MRI measurements were conducted on a whole-body 3T MRI scanner (MAGNETOM PrismaFit, Siemens Healthcare, Erlangen, Germany). Three cultivation tubes were placed into a plastic box filled with room temperature (21 °C) double-distilled water, which was secured into the head coil. The measurement protocol included a series of sequences for structural and parametric imaging.

Proton-density- and T2-weighted 2D turbo spin echo (TSE) and a T1-weighted 3D spoiled gradient echo VIBE sequences were employed for high-resolution structural imaging. To ensure precise T1 mapping, a 3D variable flip angle (VFA) approach utilizing a VIBE sequence and an additional B1-correction scan was employed. A two-dimensional Carr-Purcell-Meiboom-Gill (CPMG) multiple spin echo sequence was employed for T2 mapping. The magnetization transfer ratio (MTR) was determined through the use of a 3D spoiled gradient echo (GRE) sequence. Ultimately, a diffusion-weighted readout-segmented echo planar imaging (DWI-EPI) technique with b-values of 0, 50, 500, and 1000 s/mm^2^ and a monopolar diffusion gradient scheme was employed to quantify the apparent diffusion coefficient (ADC). The overall image acquisition took 35 min and 10 s. A summary of the imaging protocol is provided in Table 1.

**Table 1:** Imaging protocol.

	PD/T2-w	T1-w	T1-map	T2-map	DWI	MTR
Sequence	2D TSE	3D VIBE	3D VFA VIBE	2D CPMG	RESOLVE	3D GRE

TR (ms)	3000	6.8	9.2	3000	3000	25
TE (ms)	12/160	2.74	2.71	10–320	47	3.18
Flip angle (deg)	90,150	10	2/8/15	90,180	180	10
BW (Hz/Px)	191	190	190	235	744	190
Matrix	120 × 160 × 15	173 × 256x128	120 × 160 × 30	120 × 160 × 1	120 × 160 × 1	120 × 160 × 30
FOV (mm^3^)	120 × 160 × 16	116 × 154 × 38	120 × 1600 × 15	120 × 160 × 2	120 × 160 × 3	120 × 160 × 15
Voxel size Reco (mm^3^)	0.5 × 0.5 × 1.0	0.3 × 0.3 × 0.3	0.5 × 0.5 × 0.5	0.5 × 0.5 × 2.0	0.5 × 0.5 × 3.0	0.5 × 0.5 × 0.5
Voxel size Meas (mm^3^)	1.0 × 1.0 × 1.0	0.6 × 0.6 × 0.6	1.0 × 1.0 × 1.0	1.0 × 1.0 × 2.0	0.5 × 0.5 × 3.0	0.5 × 0.5 × 0.5
Scan time (min:sec)	3:41	4:28	5:37	6:05	6:53	4:48 × 2

### 2.8 Quantification and statistical data analysis

The acquired images were displayed using a self-written MATLAB code (The Mathworks Inc., Natick, Massachusetts, USA). The mapping parameters of the spheroids were determined by defining regions of interest (ROIs) and analyzed in three independent measurements. This can also be achieved using various freeware programs, including ImageJ (ImageJ, RRID: SCR_003070) and Mango (Mango, RRID: SCR_009603). Scale bars were added to the microscopic images using the ImageJ software (ImageJ, RRID: SCR_003070). The graphical and statistical analysis was conducted using an unpaired *t*-test with the software GraphPad Prism version 10.1.1 (GraphPad Software Inc., San Diego, CA, USA). Values of p < 0.05 (∗) or p < 0.01 (∗∗) were considered to be statistically significant.

## 3 Results

### 3.1 Development of a suitable setup for cell spheroids culturing and optimized conditions for MRI visualization

**Fig. 1:**
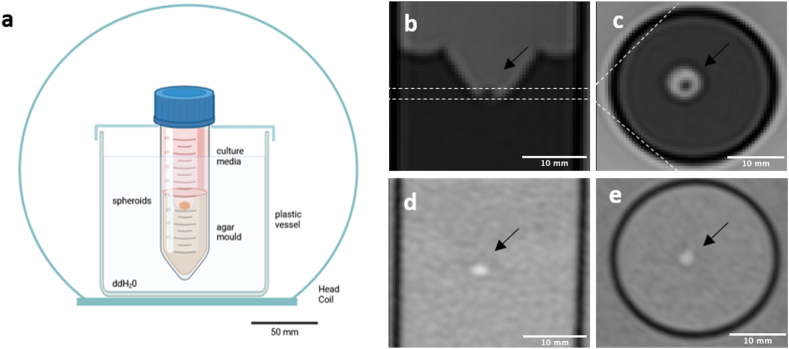
Visualization of SW1353 cell spheroids (n = 192 per tube, 62.500 cells per spheroid, indicated by arrow) in a mold of 1 % agar and culture media. Images were captured on the day of spheroid collection (day 0) to ensure immediate and accurate representation. The experimental setup was designed using Biorender.com (a), while sagittal (b) and coronal (c) T2-weighted images were obtained using an FSE sequence. Sagittal (d) and coronal (e) T1-weighted images were obtained using a spoiled gradient echo VIBE sequence. Black scale bar represents 50 mm, white scale bars 10 mm, respectively.

The experimental setup was required to meet a number of specific criteria. The cultivation conditions were created in accordance with the cells’ requirements, which entailed growth under sterile conditions with adequate ventilation and nutrient saturation. In order to prevent damage to the cells and their ECM during transfer of the spheroids, the setup also had to meet all conditions for optimal MR imaging. This excludes any electrically highly conductive components, while all of them (nutrient medium, environment, flasks) must exhibit magnetic susceptibility similar to that of water. It is essential that the external geometry of the entire test specimen is shaped spherically or cylindrically symmetrically in order to provide a homogeneous magnetic field. Furthermore, the spheroid clusters must be fixed in position and surrounded (up to a distance of approximately 1–2 cm) by a material with a low MR signal intensity and homogeneous magnetic properties. Moreover, the entirety of the sample must be accommodated within a relatively compact and uniformly distributed radiofrequency (RF) coil for signal recording purposes. In order to meet all requirements in the most optimal manner, the following structure was developed (see Materials and Methods section for details). To circumvent the potential for artefacts to arise from narrower standard flasks, a 50 ml centrifuge tube was employed for cultivation purposes. The spheroids were positioned in the center of the tube by pouring approximately half of a 1 % agarose solution and creating an indentation using a stamp. The remaining portion of the tube was then filled with the appropriate culture media. For imaging purposes, the tube was secured in a plastic box filled with double-distilled water and placed in a head coil. The proposed setup is illustrated in Fig. 1a.

### 3.2 MRI visualization of cell spheroid clusters

In order to evaluate the MRI-compatible setup, spheroids were generated from MSC and SW1353 chondrosarcoma cells, respectively. Each spheroid consisted of 62,500 cells. A total of 192 spheroids were utilized for each sample. The aggregations of spheroids were obtained on a 3T MRI scanner using structural T1-and T2-weighted imaging with a spatial resolution of 1.0 × 1.0 × 1.0 mm³ (T1) and 0.6 × 0.6 × 0.6 mm³ (T2), respectively. The T2-weighted fast spin echo (FSE) sequence demonstrates that the spheroids, agar base, and surrounding culture media can be distinctly differentiated from one another (Fig. 1b and c). The use of a T1-weighted spoiled gradient echo sequence, also known as a volumetric interpolated breath-hold examination (VIBE), revealed that the spheroids exhibited a bright signal, while the agar and media were barely discernible (Fig. 1d and e).

### 3.3 Quantitative parameter mapping along maturation of samples

**Fig. 2:**
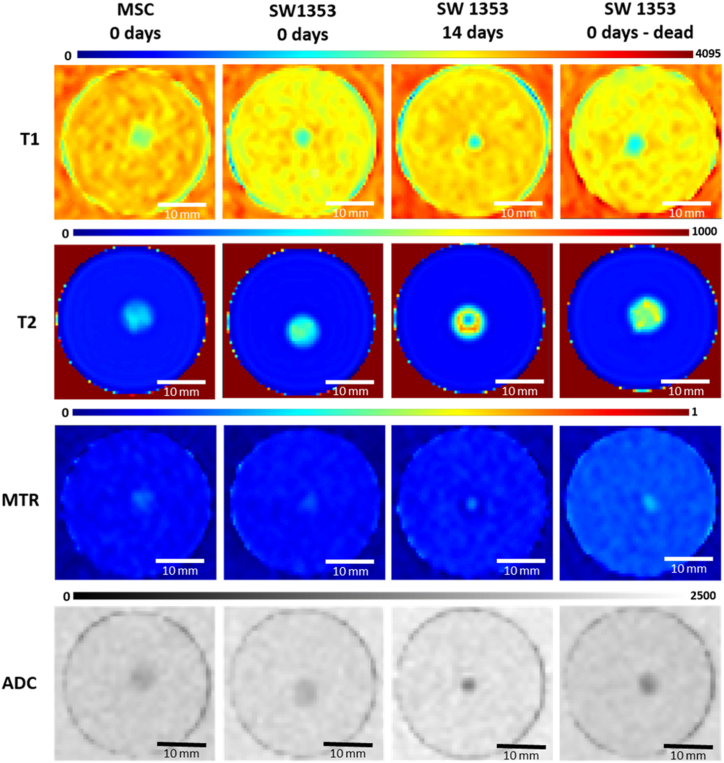
Parameter maps (T1, T2, MTR and ADC) of various cell spheroids: (MSC (1st column); SW1353 (2nd to 4th column), 0 days old, 14 days old, and dead). All samples were embedded in 1 % agar. Scale bars represent 10 mm.

**Fig. 3:**
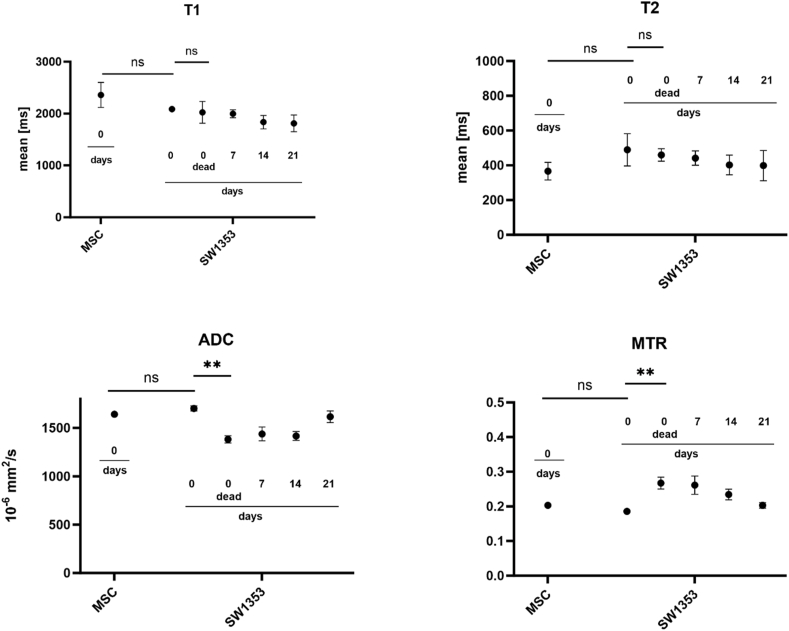
Temporal change of T1, T2, ADC and MTR values of alive and dead MSC and SW1353 cell spheroids measured over a period of 21 days. Data are represented as mean values and standard deviation from three independent experiments, ∗∗p < 0.01.

A more comprehensive cross-sectional analysis of spheroid clusters can be accomplished through a quantitative assessment of MRI features. This can be achieved through pixel-by-pixel mapping of T1 and T2 values, ADC, and MTR (Fig. 2). Such structures can be generated with both stem cells and cancer cell spheroids, as well as with identical samples over time (see Fig. 3).

The observation of T1 maps indicates that the relaxation time values for MSC are slightly longer than those for chondrosarcoma cells (MSC: 2360 ms, SW1353: 2086 ms, Table 2). If identical samples of SW1353 are measured over a 21-day period, a further decrease in the relaxation time is observed (1812 ms). Moreover, the relaxation time of dead spheroids is comparable to that of living samples of the same age (2024 ms).

**Table 2:** Acquired mean values and standard deviations for T1, T2, ADC and MTR values (n = 3) measured from MSC and SW1353 cell spheroids over a period of 21 days. Measurements were obtained from three representative defined Regions of Interest (ROI) which were all situated within the spheroid.

		T1 [ms]	T2 [ms]	ADC [10^−6^ mm^2^/_s_]	MTR
**MSC**	**0 days**	2360 ± 121	366 ± 26	1641 ± 14	0.2032 ± 0.008
**SW1353**	**0 days**	2086 ± 17	489 ± 47	1702 ± 23	0.1857 ± 0.002
	**0 days-dead**	2024 ± 39	460 ± 22	1382 ± 62	0.2672 ± 0.023
	**7 days**	1996 ± 65	442 ± 29	1438 ± 40	0.2613 ± 0.014
	**14 days**	1836 ± 81	402 ± 44	1417 ± 53	0.2345 ± 0.007
	**21 days**	1812 ± 150	398 ± 18	1616 ± 33	0.2029 ± 0.138

In contrast, the T2 relaxation times are shorter in MSCs compared to the chondrosarcoma clusters (MSC: 336 ms, SW1353: 489 ms). When observed over time, a slight decrease in the relaxation time was noted in the chondrosarcoma spheroids (day 7: 442 ms, day 21: 399 ms). The T2 relaxation times of dead chondrosarcoma spheroids are slightly shorter than those of living cells, though this difference is not statistically significant (460 ms vs. 442 ms).

A comparison of the ADC values of the two cell types reveals slightly longer values for the chondrosarcoma spheroids (MSC: 1641 × 10⁻⁶ mm^2^/s, SW1353: 1702 × 10⁻⁶ mm^2^/s). In the SW1353 spheroids, the ADC values exhibited a decline until day 14 (1417 × 10⁻⁶ mm^2^/s), followed by an increase by day 21 (1616 × 10⁻⁶ mm^2^/s). It is noteworthy that in spheroids with complete cell death, the ADC decreased significantly to 1382 × 10⁻⁶ mm^2^/s, which is markedly lower than in viable cells (p = 0.0023).

A comparison of the magnetization transfer ratio (MTR) values reveals that stem cells exhibit a similar MTR to that observed in the measured cancer cells (MSC: 0.2032, SW1353: 0.1857). This value continues to shorten over time, reaching 0.2345 by day 14. The highest MTR values are observed in dead spheroids, which exhibit a significant difference compared to viable cells (0.2672, p = 0.0091).

### 3.4 Comparing cell conditions with gold standard methodologies

**Fig. 4:**
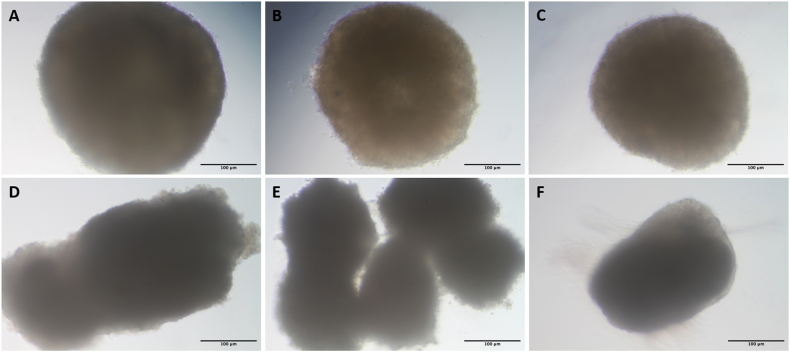
Light microscopy images of MSC and SW1353 spheroids displaying their morphology at different time points during the experiments. A: MSC at day 0, B: SW1353 at day 0, C: SW1353 at day 0 and methanol induced death, D: SW1353 at day 7, E: SW1353 at day 14, F: SW1353 at day 21. Scale bars represent 100 μm.

A comparison of spheroid morphology at different experimental time points indicates that MSC display increased cell size compared to SW1353 spheroids during the initial stages of cultivation. Following methanol treatment, the SW1353 spheroids retained their shape, although a very slight reduction in size was observed due to dehydration. Over the course of 21 days, however, the spheroids exhibited a progressive loss of structure, ultimately leading to complete disintegration (Fig. 4).

**Fig. 5:**
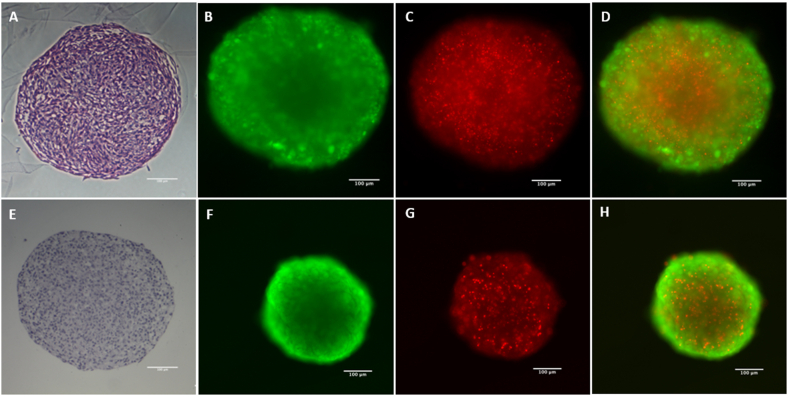
Microscopy images of MSC (upper row) and SW1353 (lower row) spheroids after five days of formation. A + E: H&E Staining, B + F: Calcein staining C + G: Propidium iodide staining, D + H: merged images of Calcein and Propidium iodide staining. Scale bars represent 100 μm.

H&E staining provides a structural overview of cell spheroids. Live/dead fluorescence staining with calcein and propidium iodide enables the visualization of discrete zones (growth, quiescent, and necrotic) within the spheroids (Fig. 5A–H). Calcein, which fluoresces green, accumulates predominantly in the outer layer, while propidium iodide accumulates in deeper cell layers. A quantitative analysis of the proportion of viable cells throughout the experimental period indicates that MSC undergo a significant decline in viability shortly after cultivation. In contrast, SW1353 cells demonstrate a notable resilience, maintaining 36 % of viable cells, even after 21 days.

## 4 Discussion

We have successfully developed an MRI setup and protocol that enables the characterization of intact samples of spheroids in follow-up studies over an extended period of time. The application of diverse MRI techniques in this method paper enables the non-invasive characterization of cell spheroids or other cell aggregations. In comparison to the preparation time required for microscopy of histological samples, the preparation time for this method is significantly reduced. The setup presented here allows for the repeated MRI study of native cell spheroids while simultaneously maintaining the appropriate cultivation conditions, thus avoiding the disruption of their spatial structure that would otherwise result from the need for translocation. The utilization of a comparatively low magnetic field strength of 3 T scanners is more favorable for spheroids and may potentially enhance the reproducibility of the setup while preserving tissue integrity more effectively than high-field imaging systems [38,39]. Our approach offers valuable insights into the status of the sample, including its viability and composition, throughout the course of cultivation. Magnetic resonance imaging (MRI) facilitates and enhances workflows by enabling rapid and repeated imaging without disrupting the structural integrity of cell spheroids, thus making it suitable for long-term culturing setups. The results were found to be stable and reproducible. In the absence of comparable studies, we established baseline values for T1, T2, ADC, and MTR parameters, thereby providing a foundational reference for future assessments.

### 4.1 MSC and chondrosarcoma spheroids reveal different characteristics

Quantitative measurements of MSC and SW1353 revealed notable differences in the intra- and/or extracellular milieus, as indicated by distinct MRI characteristics. The T1 values were higher, while the T2 values were slightly lower in MSC. One potential explanation for this discrepancy is that single-cell MSCs have a larger diameter (17–21 μm) compared to SW1353 cells (12 μm) [40]. This is also evident in our light and fluorescence microscopy images, which demonstrate that the MSC diameter is larger than that of SW1353, despite an identical cell count. In contrast with our findings, it has been published that MSC sizes decrease when cultured in spheroids [41]. It may be inferred from our results that MSC exhibited greater mortality than the chondrosarcoma cell line, likely due to their comparatively lower resilience [42,43]. There was minimal variation in ADC values. *In vivo*, malignant tissues typically exhibit reduced ADC values, which are attributed to increased cell proliferation and higher cellular densities [44,45]. However, this effect was not observed in our experimental setup. It is noteworthy that this observation was also documented by Momot et al. in their investigation of the apparent diffusivity of ovarian cancer spheroids in hydrogels [34].

### 4.2 Apparent diffusion coefficient and magnetization transfer differ in live and dead cell spheroids

A comparison of data from dead and living spheroids revealed minimal variation in T1 and T2 relaxation times in our studies, but a significant reduction in the apparent diffusion coefficient. The reduced sensitivity for cell death of transversal and longitudinal relaxation times have been shown; for instance, Valonen et al. reported a T2 increase just four days post-apoptosis induction [46], whereas Duvvuri et al. reported a rise in T1 relaxation time after six to eight days [47]. The ADC values demonstrate a notable decline upon the induction of cell death, whereas the opposite effect is observed in MTR. As a consequence of cell death, the cytoplasm is released, leaving only the scaffold of the spheroid (cell membranes and extracellular matrix) intact. This markedly diminishes the apparent diffusion of water molecules and elevates the measured proportion of macromolecules. The results of our study indicate that MTR and ADC are sensitive to the viability of young spheroids. This could prove beneficial in the future, enabling the prediction of the sequence of preclinical experiments or stem cell transplantations.

### 4.3 Changes over time may result multifactorial

The present study demonstrated the capacity to detect alterations in the intra- and extracellular environments of chondrosarcoma spheroids over the course of the incubation period. A slight reduction of T2 values and the coherent reduction of free water molecules are unlikely to be the result of a single causal factor. It is more plausible that the formation of the ECM and cell apoptosis are both involved in this process as the spheroids mature. Additionally, ADC values indicate that the data is multifactorial, as dead and seven-day-old chondrosarcoma spheroids exhibit comparable values. The diffusion of molecules can be impeded not only by the presence of dead cells but also by the accumulation of diverse macromolecules, such as the maturation of an extracellular matrix (ECM). It is noteworthy that the ADC data revert to their initial values as the spheroids mature. An increased ADC value in the later stages may be attributed to the disintegration of the spheroids, thereby facilitating the penetration of water into the tissue. This phenomenon is also discernible in the light microscopy images. A comparable pattern can be observed in the MTR data. After seven days, living spheroids exhibit values comparable to those of dead cells, gradually returning to levels akin to those of fresh tissue. To further investigate the development of the extracellular matrix (ECM), ultra-short echo-time (UTE) sequences have been demonstrated to be an appropriate tool for examining collagen-rich tissues, such as tendons [48,49].

Further research utilizing larger datasets will be essential for a more comprehensive understanding and accurate interpretation of the findings. Nevertheless, our findings demonstrate that the utilization of diverse MRI sequences and features provides supplementary insights into the composition of spheroids and the status of the sample, delivering results in a more expeditious manner in comparison to traditional histological preparations. This additional insight permits a non-destructive evaluation of newly formed microenvironments and spheroid development, thereby enhancing our understanding of their formation and progression. Our approach offers reliable insights and has the potential to serve as a significant supplement or even an alternative to existing state-of-the-art methods for monitoring cellular survival in a variety of applications.

## 5 Limitations of study

This work demonstrates that cell spheroids can be cultivated in a specialized MR-compatible container and setup, enabling visualization and non-invasive quantitative characterization with a 3T MRI scanner. Nevertheless, the predictive capacity of the results is currently constrained by the absence of reference values. Consequently, a further comprehensive comparison of results in specimens comprising different cell types with histological samples is essential. The assessment of a single spheroid is not feasible due to the limited spatial resolution of MRI on whole-body systems. To accomplish this, a specialized higher-field MR unit would be necessary, which are scarce and not readily accessible to clinical researchers.

## CRediT authorship contribution statement

**Rebecca Wißmann:** Writing – original draft, Visualization, Methodology, Investigation, Formal analysis, Data curation. **Petros Martirosian:** Writing – review & editing, Methodology, Conceptualization. **Marina Danalache:** Writing – review & editing, Supervision, Conceptualization. **Gerd Grözinger:** Resources. **Fritz Schick:** Writing – review & editing, Resources, Project administration, Methodology, Conceptualization. **Stefanie Elser:** Writing – review & editing, Project administration, Methodology, Conceptualization.

## Declaration of generative AI and AI-assisted technologies in the writing process

During the preparation of this work the authors used DeepL in order to enhance the readability of the manuscript and eliminate language issues. After using this service, the authors reviewed and edited the content as needed and take full responsibility for the content of the publication.

## Declaration of competing interest

The authors declare that they have no known competing financial interests or personal relationships that could have appeared to influence the work reported in this paper.
